# Estrogen Receptor Status Predicts Late-Onset Skeletal Recurrence in Breast Cancer Patients

**DOI:** 10.1097/MD.0000000000002909

**Published:** 2016-03-03

**Authors:** Hyun Ho Han, Sung Hwan Lee, Baek Gil Kim, Joo Hyun Lee, Suki Kang, Nam Hoon Cho

**Affiliations:** From the Brain Korea 21 Plus Project for Medical Science (HHH, JHL, NHC), Department of Pathology (HHH, BGK, JHL, SK, NHC), Department of Surgery (SHL), Severance Biomedical Science Institute (SBSI), Yonsei University College of Medicine, Seoul, South Korea (NHC), and Global 5-5-10 System Biology, Yonsei University, Seoul, South Korea (NHC).

## Abstract

Estrogen receptor-positive (ER+) breast cancer (BCa) often recurs after long latency, and is known to favor bone as a metastatic site. We hypothesized that skeletal recurrence of ER+ BCa follows a different chronological pattern from that of nonskeletal recurrence.

We retrospectively evaluated 434 matched pairs of ER+ and ER− female patients who underwent surgery for clinically localized BCa between 2005 and 2009. Patient age, tumor size, lymph node involvement, and adjuvant treatment biases were adjusted by the propensity score method. We conducted competing risk analysis to determine the prognostic significance of ER expression status on the risk of overall recurrence and late recurrence (after 3 years). We also compared chronological patterns of ER+ and ER− tumor recurrence, stratified by the first metastatic site (skeletal vs nonskeletal).

After 3 postoperative years, ER+ tumor had a significantly higher risk of overall distant recurrence than ER− tumor (*P* = 0.02). When further stratified by first site of metastasis, only late skeletal recurrence was significantly associated with ER status (*P* = 0.029). In multivariate analysis, ER and lymph node involvement status were significant prognostic factors for late skeletal recurrence, with adjusted hazard ratios of 5.2 (95% CI = 1.2–22.4, *P* = 0.025) and 5.2 (1.7–16.3, *P* = 0.005), respectively. For nonskeletal distant recurrence, tumor size (>2 cm) was the only significant risk factor with adjusted hazard ratio of 2.8 (1.4–5.7, *P* = 0.005). Annual hazard of skeletal recurrence events of ER+ tumors continued to exist up to 10 years, while annual hazard of nonskeletal recurrences decreased after peaking at 5 years. ER− tumor recurrences exhibited similar annual hazard patterns across skeletal and nonskeletal sites.

ER expression and lymph node involvement status were strong predictors of BCa late-onset (>3 years) recurrences, especially in skeletal sites. Therefore, skeletal system surveillance is mandatory for long-term follow-up of this subpopulation.

## INTRODUCTION

Metastatic recurrence is the main cause of mortality in breast cancer (BCa) patients. To prevent metastatic recurrence, chemotherapies and radiation therapies are often performed along with primary tumor surgery. However, those adjuvant therapies are effective in reducing early recurrence, but have minimal effect on the development of late recurrence.^1^ BCa metastasize most commonly to skeletal sites,^2^ which itself can lead to serious morbidities.^3,4^ Skeletal metastasis is more frequent in estrogen receptor-positive (ER+) BCa than in ER-negative (ER−).^5^ ER+ tumors are also characterized by their metastatic latency: ER+ tumors are reported to exhibit a risk for recurrence even decades after primary tumor surgery, which is very unlike the recurrence characteristics of ER-negative tumors.^6^ The aim of the present study was to evaluate the recurrence patterns of ER-positive tumors, especially focusing on the timing of the first skeletal metastasis. This information might help us understand mechanisms by which ER-positive BCa stays dormant for significantly long periods before recurrence.

## MATERIALS AND METHODS

### Study Population

Data were obtained from Severance Hospital, a single tertiary medical center in South Korea. The study was approved by the institutional review board (#4-2013-0857). All female patients with BCa diagnosis who underwent primary tumor removal surgery between 2005 and 2009 were considered for analysis. Exclusion criteria were as follows: BCa diagnosis and treatment before January 2004; preoperative or postoperative contralateral BCa; existence of distant metastases at the time of surgery; preoperative or postoperative secondary malignancy, which can mimic the metastatic recurrence of BCa; lack of clinical and pathological information on medical record; and less than 1 year of image study follow-up after surgery.

### Follow-Up, Definition of Recurrence, and Clinicopathological Factors

Mammographic surveillance, abdominal ultrasound examination, and whole body bone scan were routinely performed every 6 to 12 months within the first 5 years of follow-up, and every 12 months after that period. Additional imaging studies including positron emission tomography scan, computed tomography, and magnetic resonance imaging, were performed in specific circumstances. Distant recurrence was recorded as the primary endpoint. The site of recurrence was classified as skeletal or nonskeletal, excluding loco-regional recurrence. The clinicopathological factors of possible prognostic significance for recurrence included patient age at the time of surgery, estrogen receptor (ER), progesterone receptor (PR), human epidermal growth factor 2 receptor (HER2) expression status, histological subtype, tumor size, and lymph node involvement status. Histologic subtypes were classified as invasive ductal carcinoma, other invasive carcinoma, noninvasive ductal carcinoma in situ, and other noninvasive carcinoma. Tumor size was determined by the longest length of invasive tumor measured by pathologists or surgeons, and grouped as small (<2 cm) and large (≥2 cm). Lymph node involvement was classified as absent or present. The use of adjuvant or neoadjuvant chemotherapies, radiation, and hormonal therapies was also considered in the analysis.

### Analysis of ER, PR, and HER Expression in Tumor Tissues

The expression status of ER, PR, and HER2 was determined by immunohistochemistry of tumor tissues retrieved by surgery. A cut-off value of >1% of nuclei that were strongly stained was used to define expression of ER and PR. HER2 staining was analyzed according to guidelines by the American Society of Clinical Oncology (ASCO)/College of American Pathologists (CAP).^7^ These guidelines are as follows: a value of 0 represents no immunostaining; 1+, weak incomplete membranous staining of <10% of tumor cells; 2+, complete membranous staining, either uniform or weak, of ≥10% of tumor cells; and 3+, uniform intense membranous staining of ≥30% of tumor cells. HER2 immunostaining was considered positive when strong (3+) membranous staining was observed, while values of 0 to 1+ were considered negative. Tissues that had staining values of 2+ were further examined by fluorescent in situ hybridization (FISH) for HER-2 amplification.

### FISH

FISH was performed on tumor sections after examination by H&E microscopy. A PathVysion HER-2 DNA Probe Kit (Vysis, Downers Grove, IL) was used according to the manufacturer's instructions. The copy number of HER-2 on the slides was evaluated using an epifluorescence microscope (Olympus, Tokyo, Japan). At least 60 tumor cell nuclei in three separate regions were investigated for HER-2 and chromosome 17 signals. HER-2 gene amplification was determined according to the ASCO/CAP guidelines.^7^

### Propensity Score Matching

To compare differences in the proportions of baseline characteristics between the ER+ and ER− patient groups, χ^2^ tests and *t* test were used. To adjust the variances of other established prognostic factors and adjuvant treatment biases, the propensity score method was used. To generate a propensity score, the following parameters were used in logistic regression analysis: patient age at the time of surgery, tumor size group (2 cm), lymph node involvement status, use of chemotherapy, and use of radiation therapy. ER+ and ER− subjects were matched on a one-to-one basis, based on nearest-neighbor matching.

### Competing Risk Analysis on Distant Recurrence-Free Survival

Distant recurrence-free survival (DRFS) was determined as the duration between the date of surgery and the date of first radiological/pathological evidence of distant metastasis at any site. Follow-up times were censored on the date of last image study follow-up. Cumulative incidence of distant recurrence was calculated by the approach described by Gooley et al^8^ with death considered to be a competing event. Recurrence events were further divided by first site of recurrence, into either “skeletal” or “nonskeletal.” In this scenario, death and nonskeletal distant recurrence were considered as competing risks for skeletal recurrence, while death and skeletal recurrence were considered as competing risks for nonskeletal distant recurrence. The methods of Fine and Gray^9^ were used to compare cumulative incidence curves between ER+ and ER− patients.

### Annual Recurrence Hazard

We used the annual hazard rate (HR) to characterize chronological patterns of skeletal and nonskeletal recurrence. The annual HR of the recurrence was defined as the conditional probability of recurrence in a specific time interval, given that the patient was free of recurrence at the beginning of the interval. Series of annual HR of tumor recurrences were compared between ER+ and ER− tumors.

### Statistical Analysis

Univariate and multivariate analysis on skeletal DRFS and nonskeletal DRFS were performed using modified Cox regression model.^9^ Statistical significance was determined by Gray test and *P* values < 0.05 were considered significant. All continuous variables are presented as mean ± SD, if not specified. Statistical software SPSS 20.0 (IBM) and the R package version 2.12.1 (The R Foundation) were used for the analysis. For competing risk analysis, “cmprsk” package in R was used.

## RESULTS

### Baseline Characteristics and Recurrence Rates

A total of 1467 patients were eligible for analysis. The mean follow-up time was 76.2 ± 23.0 months. From this cohort, propensity score matching resulted in 434 matched pairs of ER+ and ER− tumors. The demographic, pathological, and treatment data of the study groups before and after matching are summarized in Table 1. Before matching, the number of ER+ subjects was 992 (67.6% of the cohort), and they exhibited significant differences in age, lymph node status, tumor size, uses of chemotherapy, radiation therapy, expressions of PR and HER2 compared to ER− subjects (Table 1). After matching, age, lymph node status, tumor size, uses of chemotherapy and radiation therapy no longer exhibited significant differences, but the PR status and HER2 status were still significantly different in the matched cohort (Table 1).

**TABLE 1:**
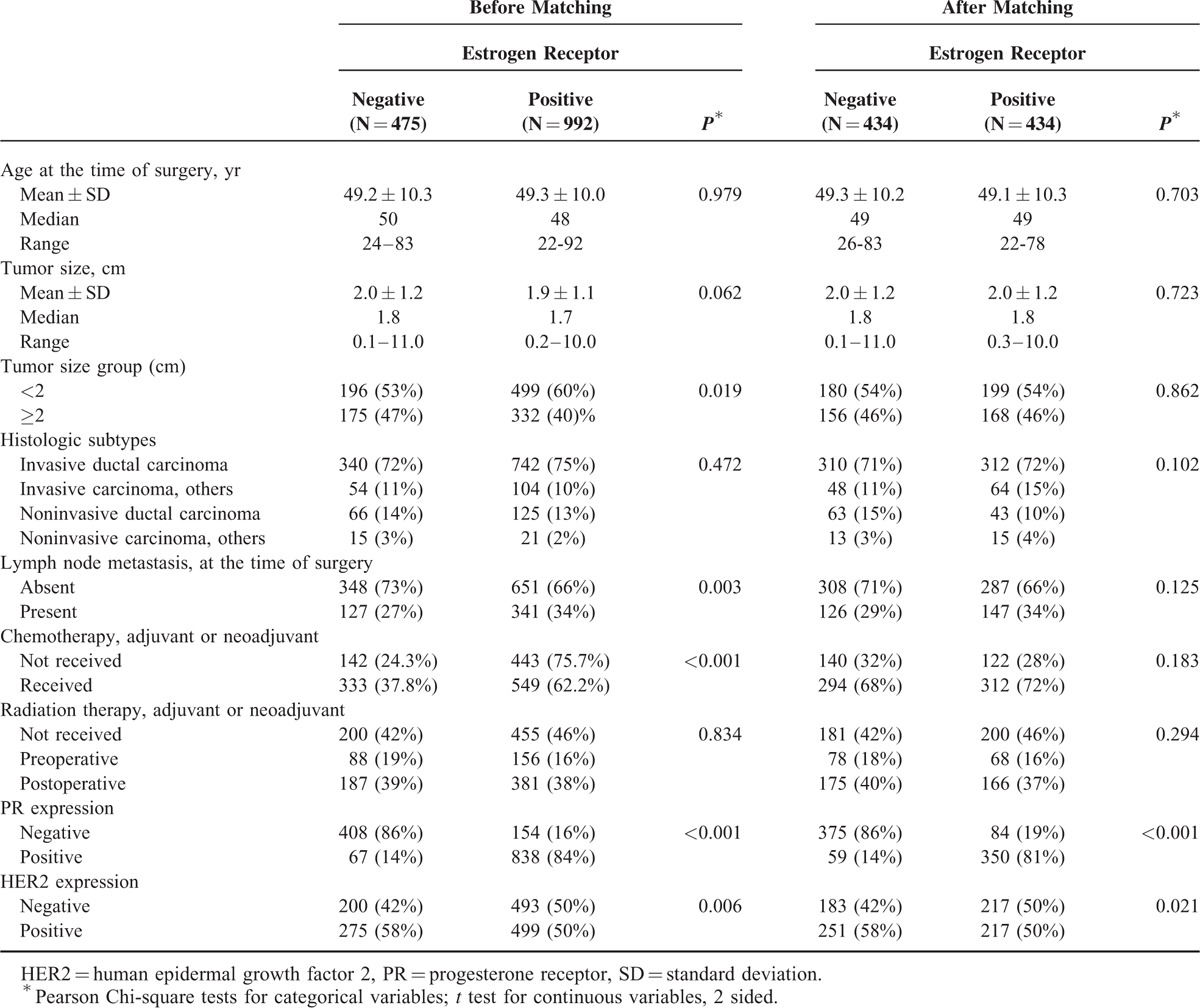
Distribution of Baseline Factors Before and After Propensity Score Matching for Patient Age, Tumor Size, Lymph Node Metastasis, Adjuvant Chemotherapies, and Adjuvant Radiation Therapies

### Impact of ER Status on Distant Recurrence-Free Survival

**FIGURE 1:**
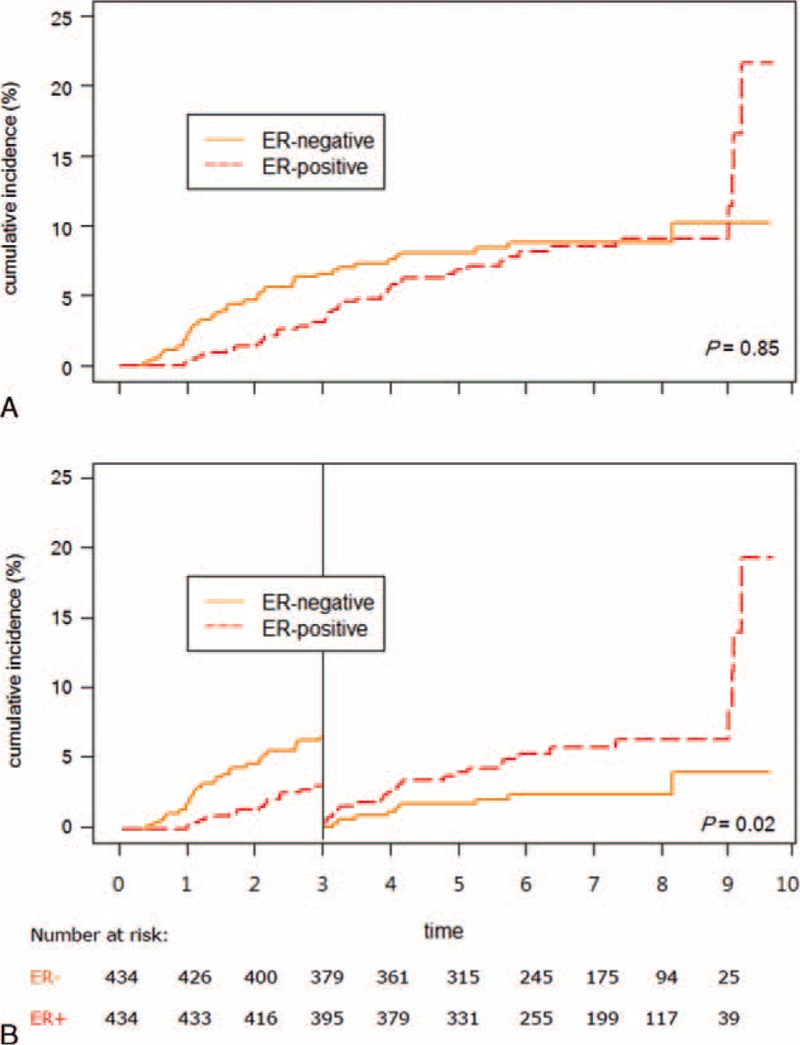
Cumulative incidences of distant recurrence in overall follow-up period (A) and three-year landmark (B), stratified by ER expression status for patients with primary breast cancer.

**FIGURE 2:**
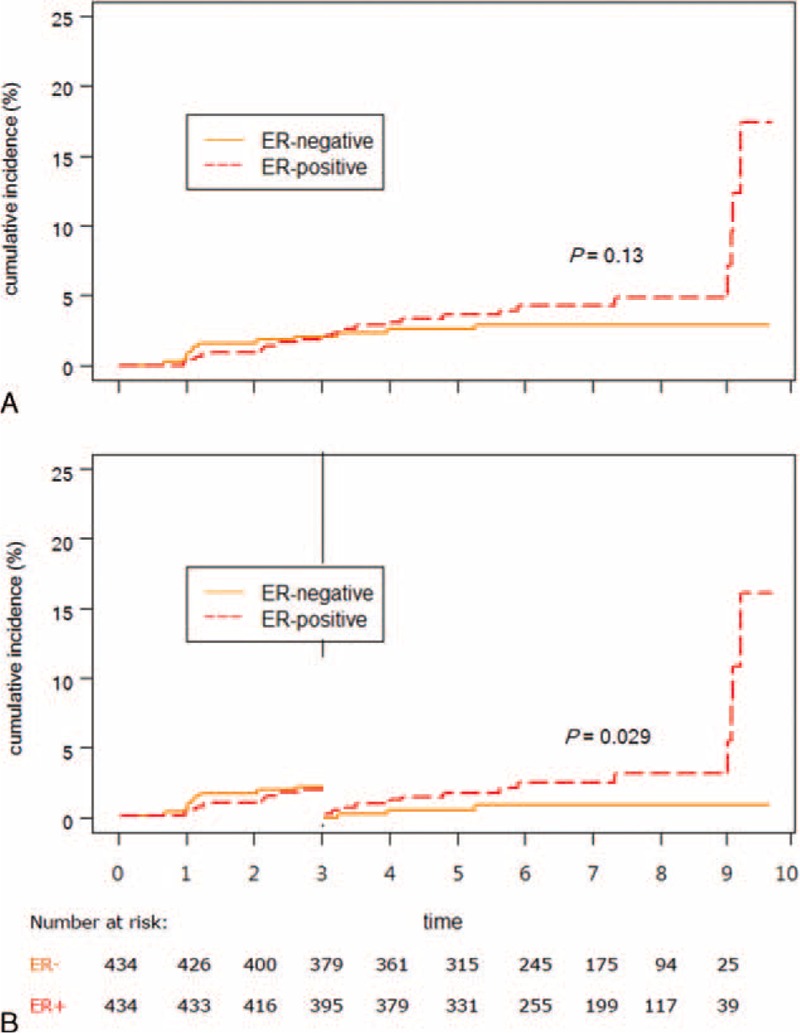
Cumulative incidences of skeletal recurrence in overall follow-up period (A) and 3-year landmark (B), stratified by ER expression status for patients with primary breast cancer.

**FIGURE 3:**
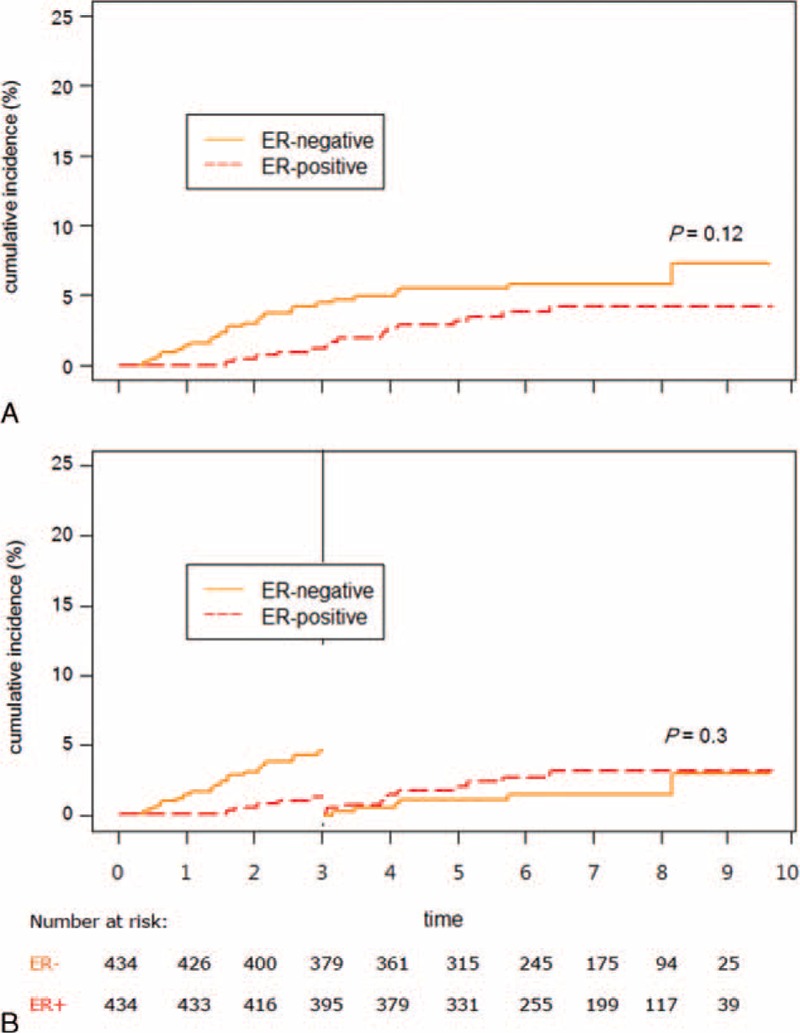
Cumulative incidences of nonskeletal distant recurrence in overall follow-up period (A) and 3-year landmark (B), stratified by ER expression status for patients with primary breast cancer.

In the matched cohort, metastatic recurrences occurred in 37 (8.5%) of ER− patients and 38 (8.8%) of ER+ patients. Five (1.2%) ER− patients and 7 (1.6%) ER+ patients died without evidence of recurrence. Competing risk analysis was performed to generate cumulative incidences of distant recurrences (Figures 1–3). Overall, the risk of distant recurrence of ER+ tumor was similar to that of ER− tumor (HR = 0.96, 95% CI = 0.61–1.5, *P* = 0.85, Figure 1A). However, confined to those who survived the initial 3 postoperative years (n = ER+ 379; ER− 361), the risk of distant recurrence was significantly higher among ER+ tumor than ER− tumors (HR = 2.48, 95% CI = 1.16–5.3, *P* = 0.02, Figure 1B).

We applied competing risk analysis separately on skeletal recurrence and nonskeletal distant recurrence. The risk of late-onset (>3 years) skeletal recurrence was significantly higher in ER+ tumor than ER- tumor (HR = 3.96, 95% CI = 1.15–13.6, *P* = 0.029, Figure 2B). In contrast, the risks of nonskeletal recurrence of ER+ tumor were similar to those of ER− tumor, both overall and late-onset (HR (95% CI) = 1.69 (0.62–4.64), 0.61 (0.33–1.15), *P* = 0.12, 0.3, respectively, Figure 3A and B).

### Factors Associated With Skeletal Recurrences

In multivariate Cox regression analysis of the matched cohort, ER status was not a predictor for skeletal recurrence overall (HR = 1.2, 95% CI = 0.7–2.3, *P* = 0.527) (Table 2). Only the established prognostic factors of lymph node involvement status was a significant predictor for recurrence, after adjustment for tumor size and the expression status of other receptors (Table 2). In those who survived the initial 3 years after surgery, however, ER positivity exhibited the highest adjusted risk for disease recurrence in the remaining period (HR = 5.2, 95% CI = 1.2–22.4, *P* = 0.025), along with lymph node involvement (HR = 5.2, 95% CI = 1.7–16.3, *P* = 0.005) (Table 3).

**TABLE 2:**
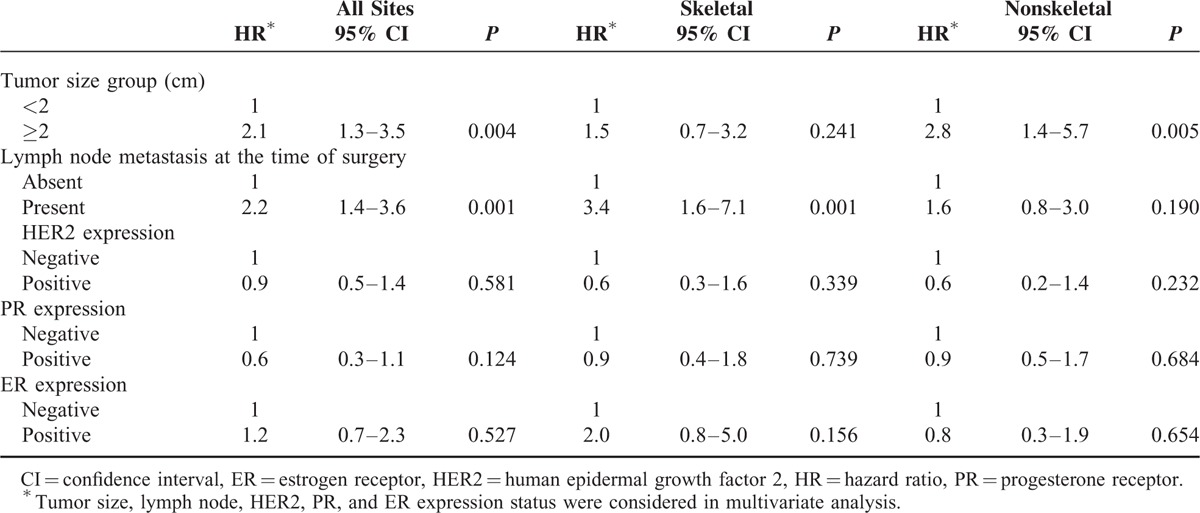
A Multivariate Cox Regression Analysis of Tumor Size Group (2 cm), Lymph Node Status, HER2, PR, and ER Expression on Distant Recurrence-Free Survival, Stratified by First Site of Distant Recurrence

**TABLE 3:**
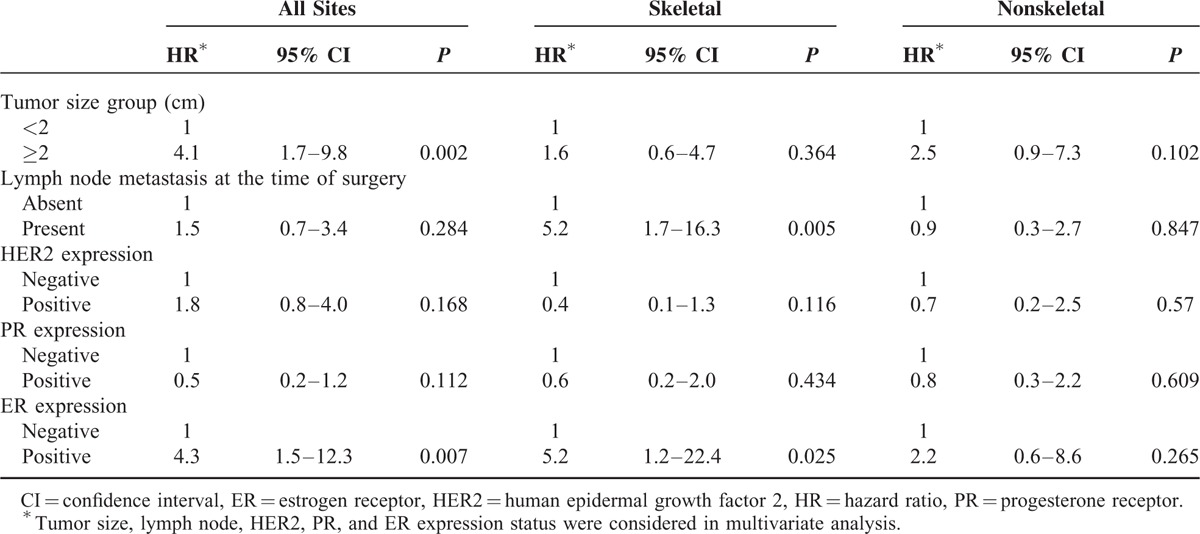
Multvariate Cox Regression Analysis of Tumor Size Group (2 cm), Lymph Node Status, HER2, PR, and ER Expression on Distant Recurrence-Free Survival in 3-Year Landmark Dataset

### Factors Associated With Nonskeletal Distant Recurrences

Multivariate Cox regression analysis showed that only tumor size (≥2 cm) was an independent risk factor of nonskeletal distant recurrence during the total follow-up period (HR = 2.8, 95% CI = 1.4–5.7, *P* = 0.005, Table 2). In those who survived the initial 3 years after surgery, neither tumor size nor lymph node involvement status were significant predictors of late-onset nonskeletal distant recurrence (Table 3).

### Recurrence Time Pattern of ER+ and ER− Tumors, Stratified by the First Site of Metastasis

**FIGURE 4:**
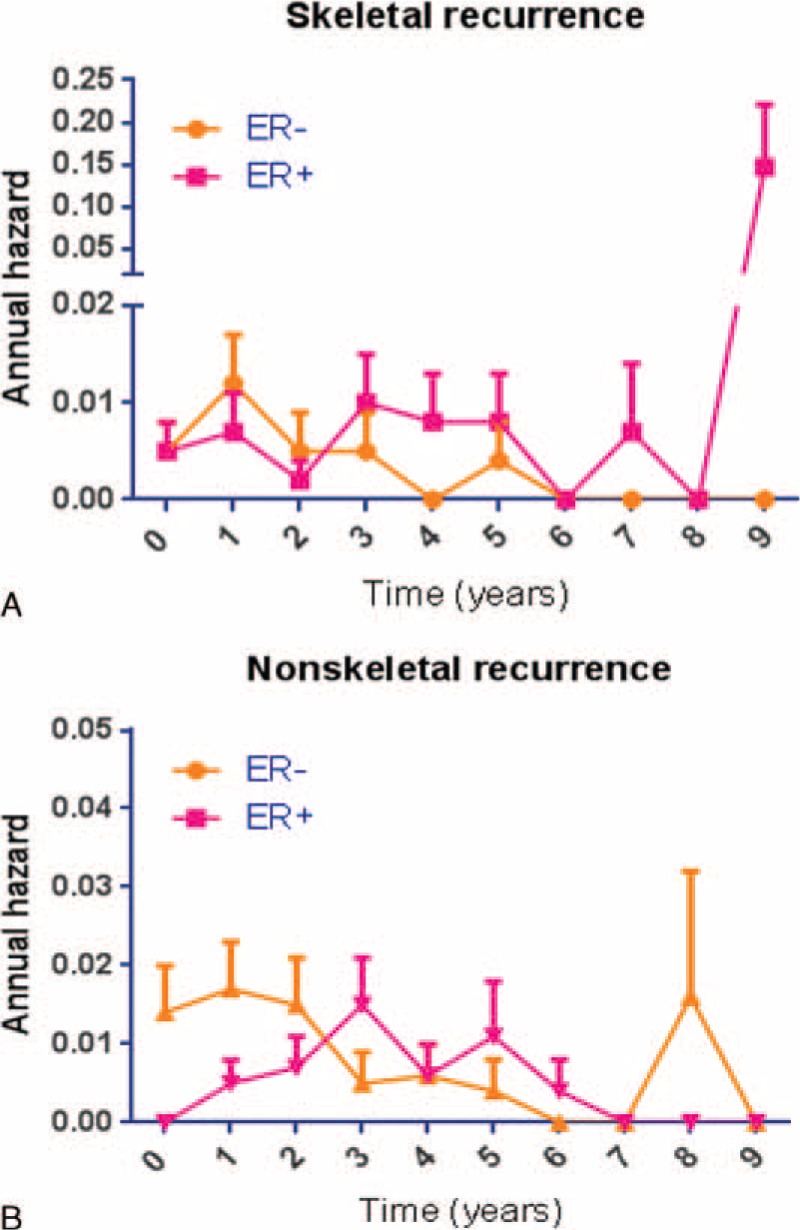
Annual recurrence hazard ratio of ER-positive and negative breast cancer patients, stratified by the first site of distant recurrence: skeletal (A), nonskeletal (B).

To determine whether ER positivity correlated with late skeletal metastatic recurrence, we calculate annual hazards of metastatic tumor recurrence time in the ER− and ER+ groups—(Figure 4). When stratified by the first site of metastasis, ER+ skeletal recurrence exhibited long-lasting risk even after 5 years. In contrast, ER− skeletal recurrences showed continuously decreasing risk right after the first postoperative year (Figure 4). Meanwhile, the annual hazard of nonskeletal recurrence in ER− tumors diminished after peaking at the first year, whereas the annual hazard of nonskeletal recurrence in ER+ tumors peaked in the fourth year and decreased gradually over the follow-up period (Figure 4).

## DISCUSSION

In this retrospective analysis, we found that ER positivity is a risk factor for the late metastatic recurrence of BCa. Many reports have produced results that are consistent with this conclusion. Earlier studies reported that more than half of ER+ tumor recurrences occur 5 years or longer after surgery for the primary tumor.^1^ Similarly, Dignam et al^10^ reported that the estimated recurrence hazard of ER+ tumors is greater than that of ER− tumors after 4 to 5 years postoperative. This long-term recurrence risk remained despite the use of the adjuvant tamoxifen and chemotherapy.

Kiba et al^6^ compared the recurrence hazard rates between ER+ and ER- BCa with axillary lymph node dissection. The hazard of recurrence for the 0- to 2-year interval was higher for ER− patients than the ER+ patients, whereas beyond 3 years, the hazard was higher for ER+ patients. This reversal of recurrence hazard around 3 to 5 years has also been noted elsewhere.^11,12^ Because our data also showed a reversal of recurrence risk between ER+ and ER− patients around 3 years postoperatively, we performed multivariate Cox regression analysis for overall follow-up period and also specifically for 3 years and after. We found that ER status acts as a DRFS indicator after 3 years, in which nodal status was not a significant factor for recurrence, and only the tumor size (2 cm or higher) was significant in both time frames. Interestingly, when stratified by first site of metastasis, tumor size is associated not with skeletal recurrence but only with nonskeletal recurrence for overall follow-up period. Meanwhile, lymph node involvement is associated only with skeletal recurrence, not with nonskeletal recurrence, both overall and late-onset.

Skeletal metastasis and nonskeletal metastasis have different impacts on prognosis. Patients with bone metastases at first relapse have better overall survival than patients with visceral metastases at first relapse.^13,14^ Furthermore, bone metastases usually develop later than visceral metastases (median, 33 months vs 26 months).^13^ Our results add significance to these previous findings by analyzing the recurrence time separately in early (<3 years) and late (>3 years) postoperative periods. Nonskeletal metastases in ER+ tumors developed mostly around 5 years postoperatively, which is about 2 to 3 years later than for ER− tumors. In contrast, skeletal metastases in ER+ tumors were frequent around 8 to 10 years postoperatively, while in ER− tumors skeletal metastasis were rare after 5 years. The pattern of ER− tumor recurrence observed in our study is in line with that seen in a study by Dent et al.^15^ They classified the site of first recurrence as visceral and bone, and found that a triple-negative tumor group exhibited similar or lower risk of bone recurrence during 0 to 5 years of follow-up, while exhibiting significantly higher risk of visceral recurrence in the same period.^15^ After 5 years, the risk of both bone and visceral recurrences in the triple-negative group was significantly lower than for the other forms of BCa. Together with those previous results, our observation emphasizes the importance of follow-up protocol optimization according to the time period and the biological characteristics of the tumor. For instance, we suggest more frequent surveillance in skeletal system (by bone scan) in patients with ER+ BCa with positive lymph node involvement, especially after 3 postoperative years. We also suggest more frequent abdominopelvic imaging study in patients with primary tumor size ≥2 cm. Such individualization in follow-up monitoring will increase the efficiency and most importantly help detecting metastatic recurrence as early as possible.

In this study, we focused on the “first” metastatic recurrence event, not on the whole metastatic events. In other words, all recurrence events in our study were divided into either “skeletal” or “nonskeletal” groups without overlapping. For example, if a patient developed bone metastasis at 5 years postoperatively and lung metastasis at 6 years, we counted this case as “skeletal metastasis, 5 years”, not as “skeletal metastasis, 5 years, plus nonskeletal metastasis, 6 years.” This is why we considered the occurrence of skeletal recurrence as a competing risk of nonskeletal recurrence, and vice versa. The rationales of our discrimination method “first metastatic recurrence” are as follows. In clinic, if a metastatic tumor is detected during routine postoperative follow-up, then additional imaging studies are usually followed to find out possible concurrent metastasis in other sites. Moreover, a patient may undergo salvage chemotherapy or else to treat the first metastatic event, which can hinder the occurrence of subsequent metastatic recurrences. In addition, in the example, subsequently noticed lung metastasis may have originated from primary breast tumor; or (2) metastatic bone tumor. The possibility of secondary metastasis was also of our concern that made us to focus on “first” event of metastasis. Since our study was designed in retrospective manner, it was impossible to control those variations. We believe a prospectively designed cohort can overcome such limitations.

Above all, the late metastatic recurrence of ER+ BCa predominant in bone brings us the clinical and scientific challenges of tumor dormancy. Multiple mechanisms have been proposed to explain how cancer cells survive and remain in dormancy, and how they become reactivated and exit dormancy, including angiogenic switch, immunosurveillance, and interaction with extracellular matrix and stromal cells.^16–18^ In particular, research has shown that these dormant breast tumor cells reside preferentially in bone marrow, and eventually become the source of metastatic recurrence.^19,20^ Clinically, the presence of disseminated tumor cells (DTCs) in bone marrow is a predictor for late metastatic recurrence.^21,22^ However, the exact factor that triggers metastatic reactivation in bone has not been identified yet. Recently, Ghajar et al^23^ reported that bone marrow and pulmonary microvascular instability promotes the proliferation of previously quiescent breast tumor cells. Meanwhile, Ottewell et al^24^ reported that experimental ovariectomy enhanced bone resorption and induced the growth of DTCs in bone. We suggest that such changes in the bone microenvironment may be monitored, or even prevented, to specifically suppress late bone metastatic recurrence of ER+ tumors, because many patients are at risk for osteoporosis and vascular accident after menopause induced naturally, by estrogen block or even by chemotherapy-induced ovarian failure.

A limitation of this study is that adjuvant therapy guidelines have changed during the time period of this cohort. Around 2005 to 2008, the adjuvant chemotherapeutic regimen was largely converted from conventional CMF (cyclophosphamide, methotrexate, fluorouracil) and anthracycline-based combinations, to taxanes, gemcitabines, and biological agents such as trastuzumab and bevacizumab.^25–27^ Furthermore, new generation aromatase inhibitors have shown to be effective in addition to tamoxifen.^28^ Based on this, many of our latter patients were treated with modern chemotherapeutics and aromatase inhibitors. The relatively overall low recurrence rates of our studies compared to previous results could be explained by such changes in adjuvant treatment options.

We used propensity score matching to adjust the deviations in patient age, tumor size, lymph node status, chemotherapy and radiation therapy use in the ER+ and ER− patient groups, but whether the statistical analogy was appropriate to generate the conclusion is debatable. Along with ER, hormonal receptors PR and HER2 can also affect the outcome of BCa. However, since ER is often coexpressed with those receptors in BCa tissue, including those would result in significant shrinkage of matched cohort. A comparative analysis of distant recurrence pattern among triple negative, luminal, and HER2-expressing subtypes will be less prone to those receptor confounders.

## CONCLUSION

ER expression status, along with lymph node involvement, predicted late-onset (>3 years) skeletal recurrences of BCa. The relationship between tumor characteristics and the likelihood of late metastatic disease demonstrates the need of more sophisticated approaches to long-term follow-up monitoring to improve management.
